# Supplementary material for: Effect of nonsense-mediated mRNA decay factor SMG9 deficiency on premature aging in zebrafish
Source: Commun Biol. 2024 May 28;7:654. doi: 10.1038/s42003-024-06356-6 (PMC11133409; doi:10.1038/s42003-024-06356-6)

Supplementary Information

Effect of nonsense-mediated mRNA decay factor SMG9 deficiency on premature aging in zebrafish

Shaohong Lai[a,1], Hiroshi Shiraishi[a,1], Wulan Apridita Sebastian[b], Nobuyuki Shimizu[a], Ryohei Umeda[c], Mayo Ikeuchi[a], Kyoko Kiyota[a], Takashi Takeno[a], Shuya Miyazaki[a], Shinji Yano[d], Tatsuo Shimada[e], Akihiko Yoshimura[f], Reiko Hanada[c], and Toshikatsu Hanada[a,*]

[a]Department of Cell Biology, Oita University Faculty of Medicine, Yufu, Oita, Japan

[b]Department of Pediatrics, Oita University Faculty of Medicine, Yufu, Oita, Japan

[c]Department of Neurophysiology, Oita University Faculty of Medicine, Yufu, Oita, Japan

[d]Institute for Research Management, Oita University, Yufu, Oita, Japan

[e]Oita Medical Technology School, Japan College of Judo-Therapy, Acupuncture & Moxibustion Therapy, Oita, Japan.

[f]Department of Microbiology and Immunology, Keio University School of Medicine, Tokyo, Japan.

[1]Lai and Shiraishi contributed equally to this work.

*Corresponding author E-mail: thanada@oita-u.ac.jp

Supplementary Table 1. List of oligo primers used in this study

Primer	Forward	Reverse
**For qPCR**		
rpl22l1 (Danio rerio)	CCAAGATGGCACCGAAAAGG	TTGCACTACATTGCCCAGGT
srsf3a (Danio rerio)	TGATTATAGACGCCGCAGCA	TGGATCGCGAACGACTGC
srsf5a (Danio rerio)	TGGCGGTTCCAGGTATGGAC	CACTGTGTGAAGCAAACTCCA
rpl10a (Danio rerio)	TCACGCCAAAATGAGCAAGG	GGTCTCCAAGAACGCAGACA
gadd45b (Danio rerio)	CACTGCATCCTCGTCACTAACT	CCATTGGTTTTTGCAACGGC
atf4 (Danio rerio)	GATGAGGAGAGCTCCGTGTTG	GAAACACATCGCCTGCTGTGC
pdrg1 (Danio rerio)	CCGTCTCAATGAGCTACAAGGTAA	CCAGGCTCAAAGCAGTCTGA
cars (Danio rerio)	CAGGAGACGCAGCCTTTGA	CCAGGTAACTGCGACTGCTC
ddit3 (Danio rerio)	CTGATTGGTGCGATGACTGC	ACTCGGGCTCCTTCTCTGAA
rassf1 (Danio rerio)	AACCGGGGAAGTGAATTGGG	TGCCGGACGTGATCTTCTTC
p21-cdkn1a (Danio rerio)	CGCAAACAGACCAACATCAC	AACGCTGCTACGAGACGAAT
p27-cdkn1bb (Danio rerio)	TGAAGCCTGGAACTTCGACT	TGTGAATATCGGAGCCCTTC
p57-cdkn1ca (Danio rerio)	TGAGATGAAACGCAAACTGC	CCTCCCACTCGTAATCTCCA
p16-cdkn2a/b (Danio rerio)	TGAACGTCGAGGATGAACTG	AAGGTGCGTTACCCATCATC
p53-tp53 (Danio rerio)	GATGGTGAAGGACGAAGGAA	AAATGACCCCTGTGACAAGC
gpx3 (Danio rerio)	ACCATCCTCGGCTTTCCTTG	AGGCGGACATGCGTTCTTTA
osbp2 (Danio rerio)	CCACACTCTTCCGCATCACT	CCTCTGCTGCTTGGTTGAGT
smox (Danio rerio)	TAATGGCAAGCGAATCCCGA	TTCACAGCTCTCCACCTTGA
actb1 (Danio rerio)	CGAGCTGTCTTCCCATCCA	TCACCAACGTAGCTGTCTTTCTG
il-6 (Danio rerio)	TCAACTTCTCCAGCGTGATG	TCTTTCCCTCTTTTCCTCCTG
il-1β (Danio rerio)	TTCCCCAAGTGCTGCTTATT	AAGTTAAAACCGCTGTGGTCA
tnfα (Danio rerio)	ACCAGGCCTTTTCTTCAGGT	GCATGGCTCATAAGCACTTGTT
smg9 (Danio rerio)	CAGTCAGCCCGGTCTGTAC	TGGATCTCTCTCCAGGAGGT
smg8 (Danio rerio)	GACACTGACAGTCCGGGC	CAAAACTCGTGGGCTTCTGC
**For genotyping**		
smg9 (HMA)*	GTGTCAGCATGTCGGAGTCC	GAGAGGTTTTGACCAGGGGG
smg8 (HMA)*	GCAAATAAACACATCTTCTCTCTCTT	ATACGCCTGTATTGCAGCTC

*HMA: heteroduplex mobility assay

Supplementary Table 2. List of gene abbreviation, full names and functions in this study

Gene	Gene name	Gene function
SMOX	Spermine Oxidase	The excitotoxic damage and increased oxidative stress associated with neurodegenerative diseases
GPX3	Glutathione Peroxidase	Contributing to cellular health and protection against oxidative stress
OSBP2	Oxysterol Binding Protein 2	Essential for cell proliferation and survival and is involved in maintaining cell function and vitality
SRSF	The Serine/Arginine-rich Splicing Factor	Promote the inclusion of exons and removal of introns during splicing
GADD45B	Growth Arrest and DNA Damage-Inducible Protein 45 Beta	Responses to various stresses in cellular, including DNA damage and growth arrest
CARS	Cysteinyl-tRNA synthetase	An enzyme links cysteine to its matching tRNA, ensuring its inclusion in growing protein chains during translation
DDIT3	DNA Damage Inducible Transcript 3	Response to DNA damage, endoplasmic reticulum (ER) stress, and various environmental stresses in cellular
PDRG1	P53 and DNA Damage Regulated 1	Associated with the regulation of the tumor suppressor protein p53 and is involved in cellular responses to DNA damage
ATF4	Activating Transcription Factor 4	A crucial transcription factor that responds to cellular stress and orchestrates adaptive responses to maintain cellular homeostasis. Its role in amino acid metabolism, ER stress response
RASSF1	Ras Association Domain Family Member 1	Regulating cell growth, apoptosis (programmed cell death), and other cellular processes as a tumor suppressor gene
RPL22L1	Ribosomal Protein L22 like 1	Component of the ribosome for stability and facilitating peptide bond formation during protein synthesis
RPL10a	Ribosomal Protein L10a	Component of the ribosome assembly that catalyzes peptide bond formation in protein

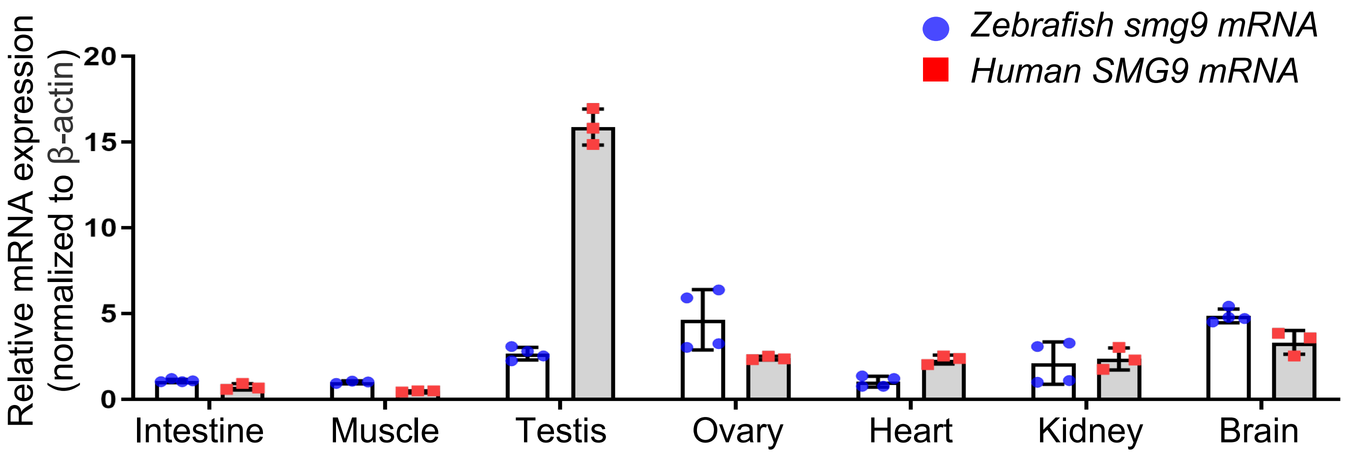

Supplementary Figure 1. RT-qPCR analysis of *SMG9* mRNA levels in zebrafish and human tissues.

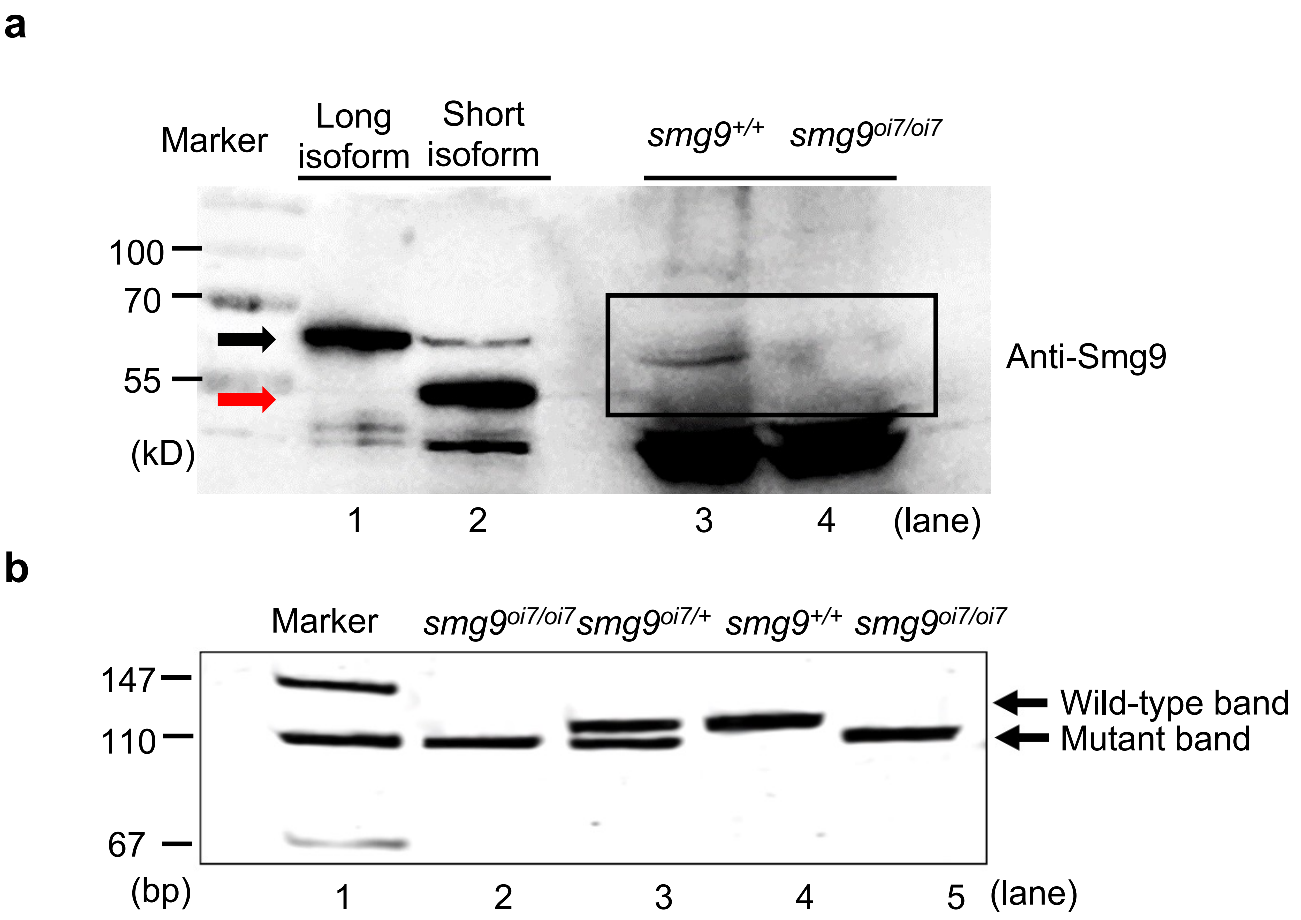

Supplementary Figure 2. **(a)** Western blot analysis of Smg9 protein in zebrafish testes. Lane 1 shows the long isoform of 3xFLAG-tagged recombinant Smg9 (black arrow); lane 2 shows the short isoform of 3xFLAG-tagged recombinant Smg9 (red arrow). The long isoform of Smg9 was observed in wild-type zebrafish (lane 3). Smg9 was not detected in the *smg9*$^{oi7/oi7}$ zebrafish (lane 4). The short isoform was absent in both the wild-type and *smg9*$^{oi7/oi7}$ zebrafish. **(b)** PCR genotyping of *smg9*$^{oi7/oi7}$ zebrafish. Genotyping results identifying *smg9*$^{+/+}$ and *smg9*$^{oi7/oi7}$. Genomic DNA from the tails of second-generation (F2) zebrafish was amplified using PCR. The first lane shows the DNA marker, and lanes 2 and 5 indicate homozygotes (*smg9*$^{oi7/oi7}$). Lane 3 indicates a heterozygote (*smg9*$^{oi7/+}$), and lane 4 indicates the wild-type (*smg9*$^{+/+}$). kD: kilodalton; bp: base pair.

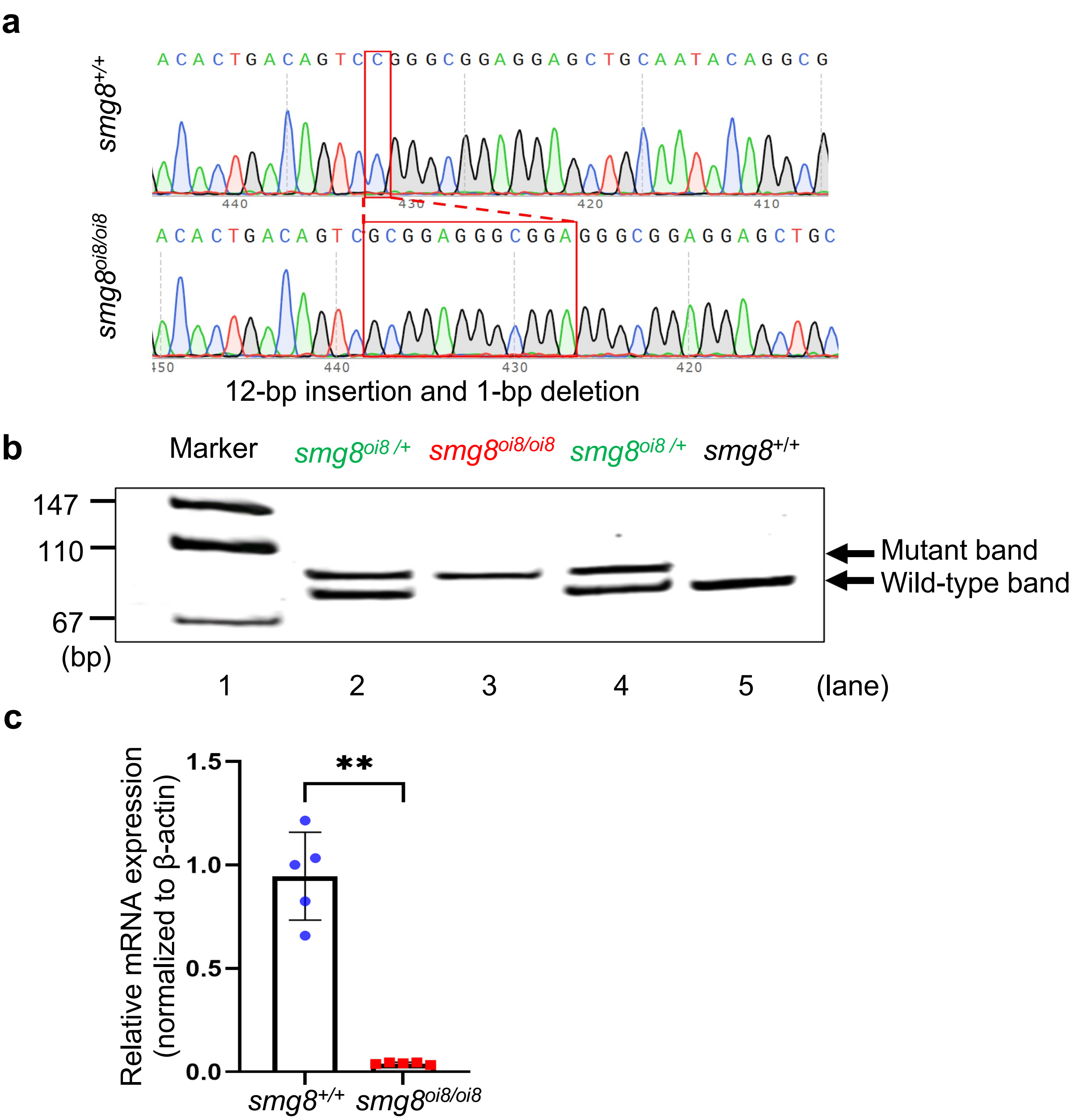

Supplementary Figure 3. Schematic illustration of generating *smg8*$^{oi8/oi8}$ zebrafish. (a) Genomic locus diagram depicting the *smg8* gene and CRISPR/Cas9-induced insertion of 12 bp and deletion of 1 bp in zebrafish, resulting in an *smg8* frameshift mutation. The red box indicates the mutated region in *smg8*$^{oi8/oi8}$ zebrafish. **(b)** Genotyping results for the *smg8*$^{+/+}$ and *smg8*$^{oi8/oi8}$ zebrafish. The first lane shows the DNA markers. Lanes 2 and 4 indicate heterozygotes (*smg8*$^{oi8/+}$). Lane 3 indicates a homozygote (*smg8*$^{oi8/oi8}$), and lane 5 indicates wild-type (*smg8*$^{+/+}$). **(c)** RT-qPCR analysis of *smg8* mRNA expression in the *smg8*$^{+/+}$ and *smg8*$^{oi8/oi8}$ zebrafish at 6 mpf. Error bars indicate the standard deviations (SD). $**P < 0.01$.

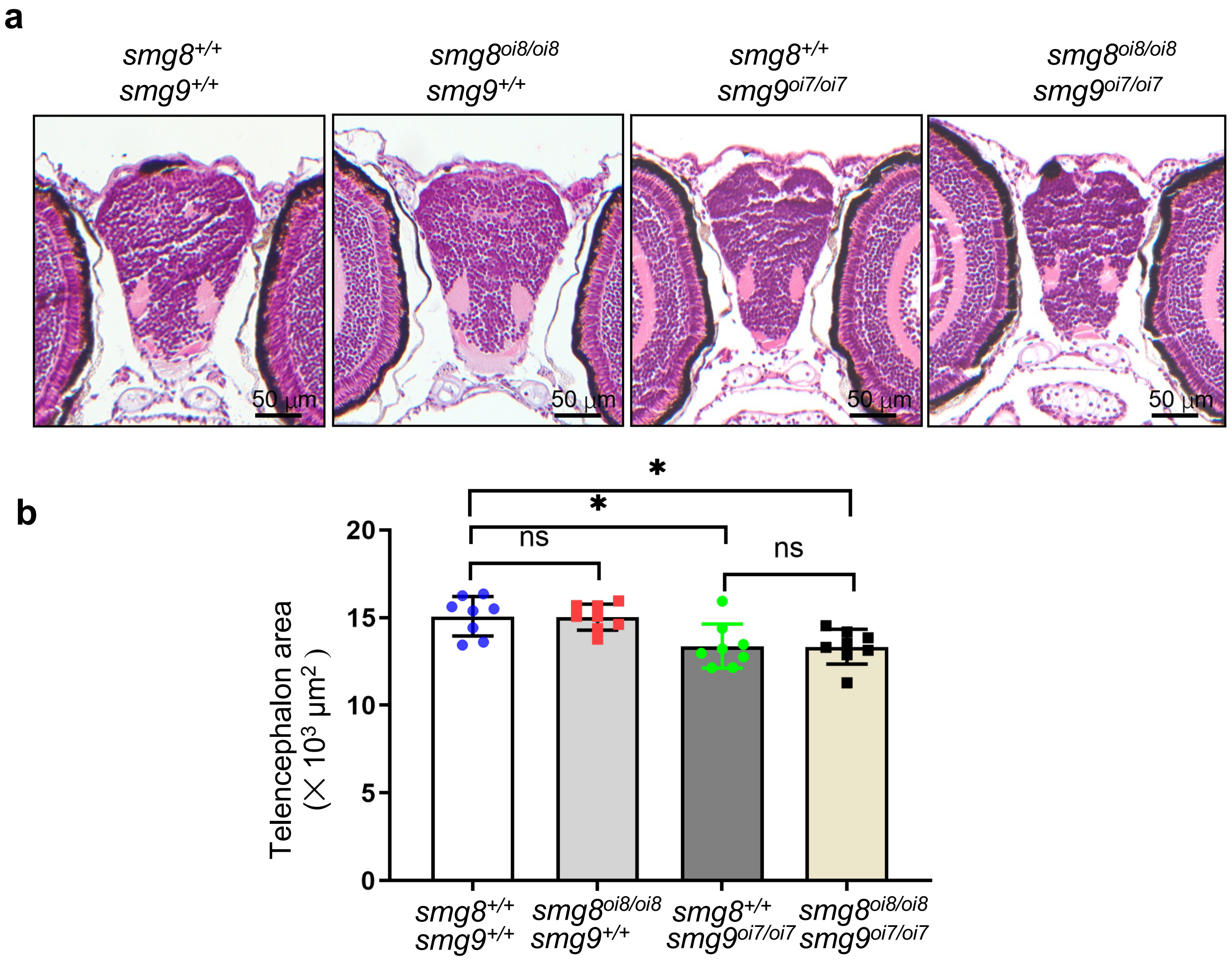

Supplementary Figure 4. Comparison of brain size in single and double SMG mutant zebrafish. (a) Representative images of hematoxylin and eosin (H&E) staining of telencephalons of *smg9*$^{oi7/oi7}$, *smg8*$^{oi8/oi8}$, and double mutant (*smg8*$^{oi8/oi8}$/*smg9*$^{oi7/oi7}$) larvae at 14 days post-fertilization (dpf). Scale bar: 50 µm. **(b)** Quantification of the telencephalon area of *smg9*$^{oi7/oi7}$, *smg8*$^{oi8/oi8}$, and double mutant (*smg8*$^{oi8/oi8}$/*smg9*$^{oi7/oi7}$) larvae at 14 dpf. Error bars indicate SD. *$P < 0.05$. ns: not significant.

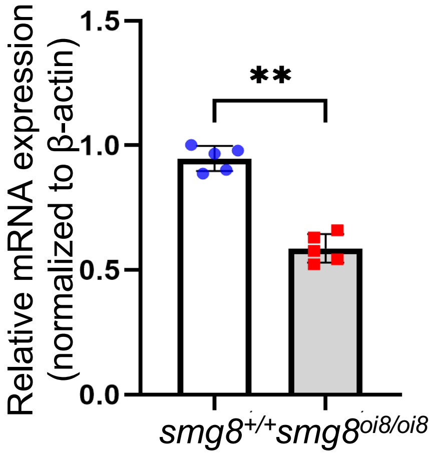

Supplementary Figure 5. Potential compensatory mechanism between Smg8 and Smg9. Quantitative PCR analysis of *smg9* mRNA expression in the $smg8^{+/+}$ and $smg8^{oi8/oi8}$ zebrafish at 6 mpf. Error bars indicate SD. $^{**}P < 0.01$.

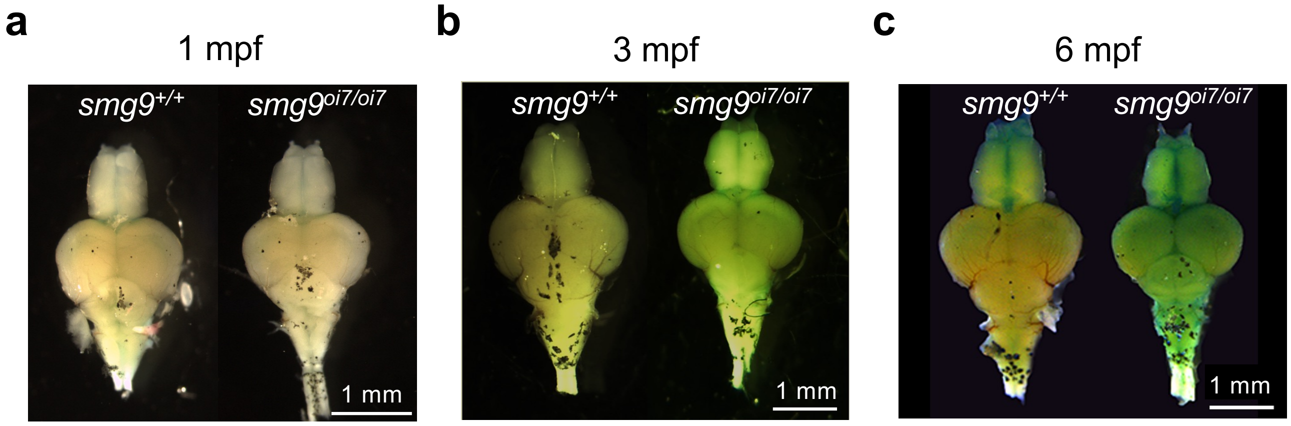

Supplementary Figure 6. Premature aging phenotypes of the *smg9*$^{oi7/oi7}$ zebrafish. SA-β-gal staining of the brains of *smg9*$^{+/+}$ and *smg9*$^{oi7/oi7}$ zebrafish at 1 mpf (a), 3 mpf (b), and 6 mpf (c). Scale bar: 1 mm.

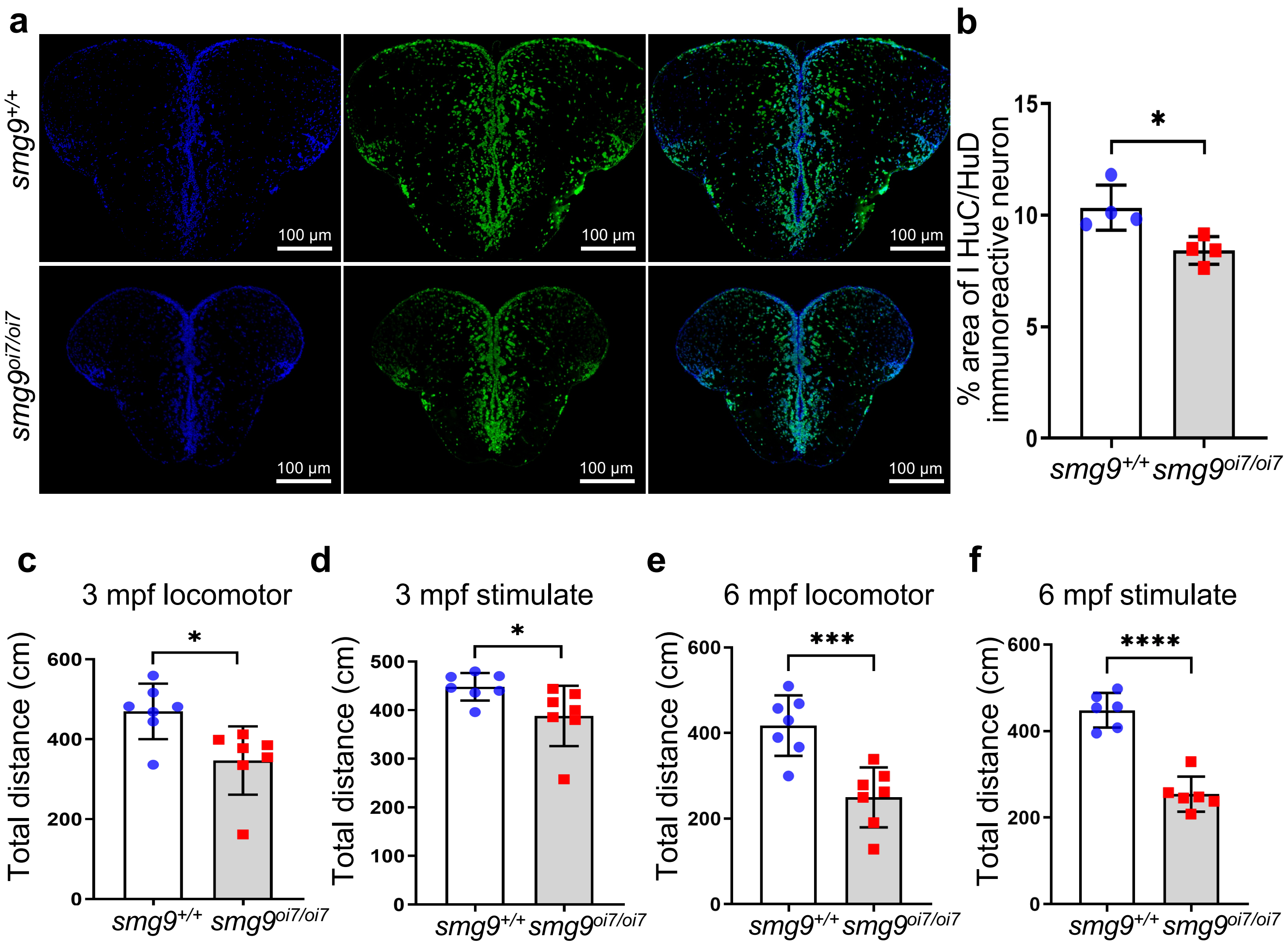

Supplementary Figure 7. Decreased mature neurons and locomotor activity in *smg9^oi7/oi7^* zebrafish. (a) Representative images of immunostaining of the brain using HuC/HuD and co-staining with DAPI in the $smg9^{+/+}$ and $smg9^{oi7/oi7}$ zebrafish at 6 mpf. Scale bar: 100 µm. **(b)** Quantification of the %area of HuC/HuD-immunoreactive mature neurons in the $smg9^{+/+}$ and $smg9^{oi7/oi7}$ zebrafish at 6 mpf. **(c, d)** Locomotor activity and acoustic stimulation in $smg9^{+/+}$ and $smg9^{oi7/oi7}$ zebrafish at 3 mpf. **(e, f)** Locomotor activity and acoustic stimulation in $smg9^{+/+}$ and $smg9^{oi7/oi7}$ zebrafish at 6 mpf. Error bars indicate SD. $*P < 0.05$, $**P < 0.01$, and $***P < 0.001$.

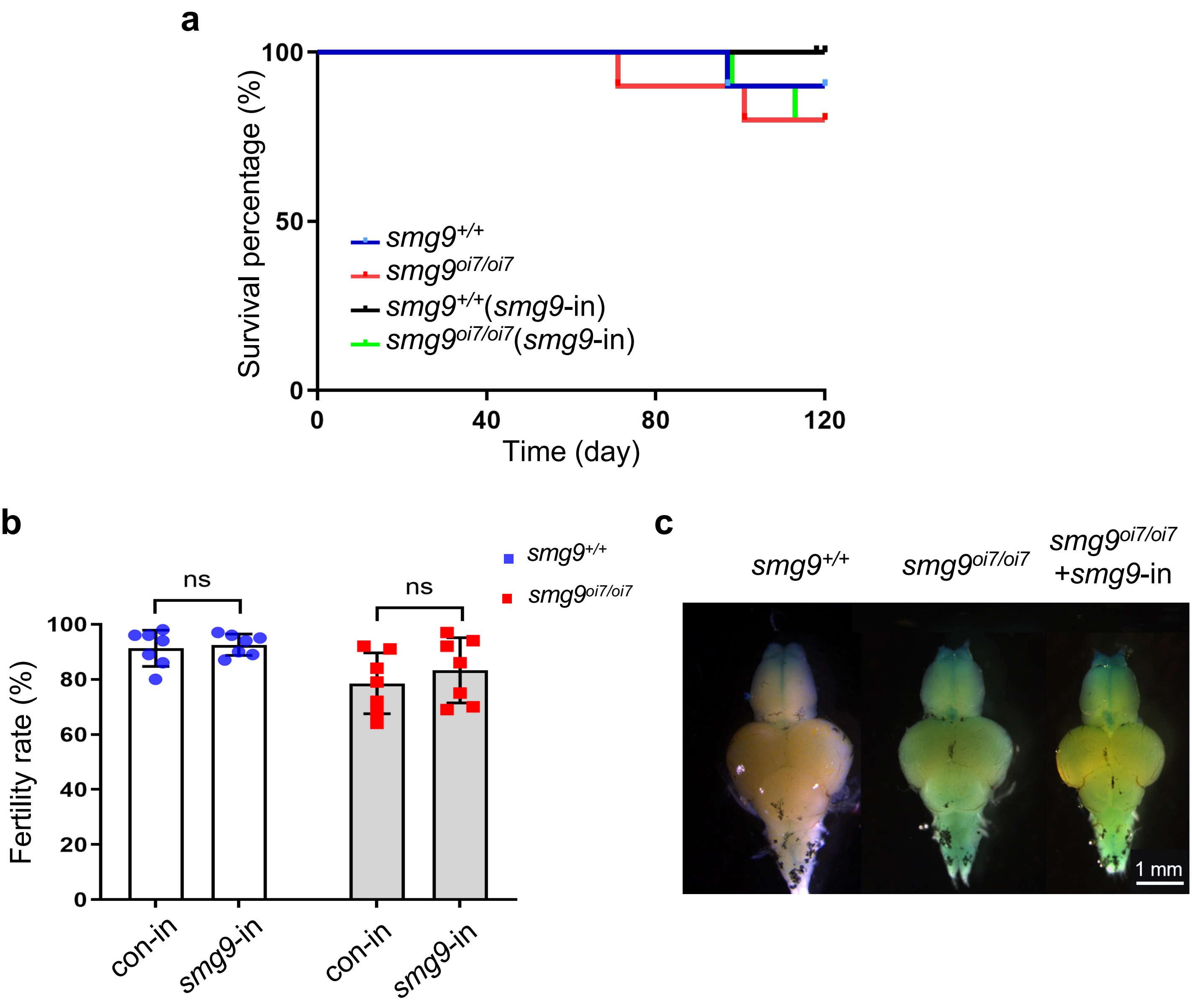

Supplementary Figure 8. Injection of smg9 mRNA (smg9-in) and control (con-in) into *smg9*$^{+/+}$ and *smg9*$^{oi7/oi7}$ zebrafish. (a) Kaplan–Meier survival curve analysis showing the lifespan of *smg9*$^{+/+}$ and *smg9*$^{oi7/oi7}$ zebrafish, each group (n =10). (**b**) Fertility rate of *smg9*$^{+/+}$ and *smg9*$^{oi7/oi7}$ zebrafish at 3 mpf, as determined by mating *smg9*$^{+/+}$ and *smg9*$^{oi7/oi7}$ male zebrafish with *smg9* female zebrafish and counting embryo survival. (**c**) SA-β-gal staining of brains of *smg9*$^{+/+}$ and *smg9*$^{oi7/oi7}$ zebrafish at 4 mpf.

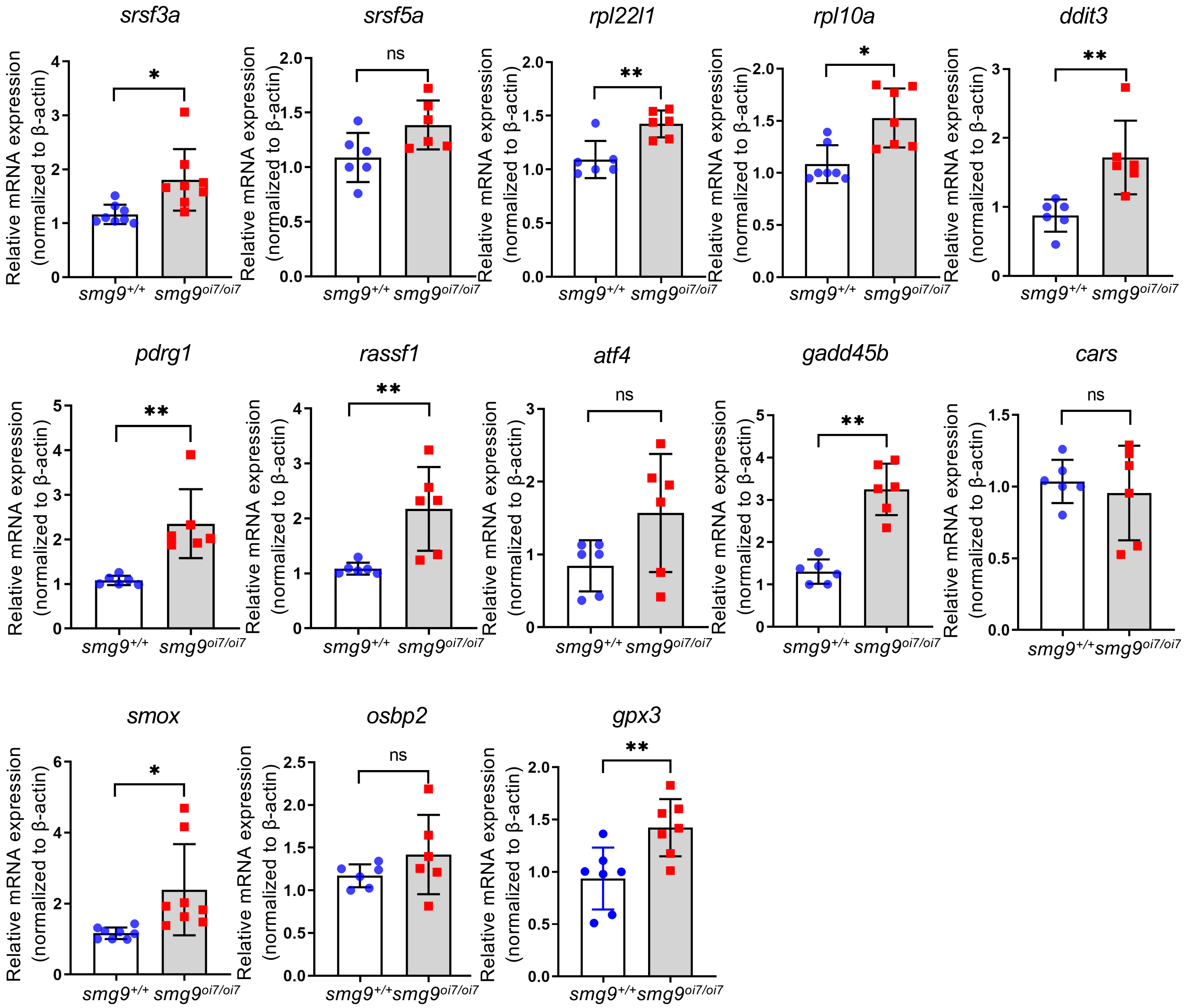

Supplementary Figure 9. Upregulation of endogenous NMD targets in adult *smg9*$^{oi7/oi7}$ zebrafish. RT-qPCR analysis of endogenous target genes of NMD in *smg9*$^{+/+}$ and *smg9*$^{oi7/oi7}$ zebrafish at 6 mpf. Error bars indicate SD. **P* < 0.05, ***P* < 0.01, and ****P* < 0.001. ns: not significant.

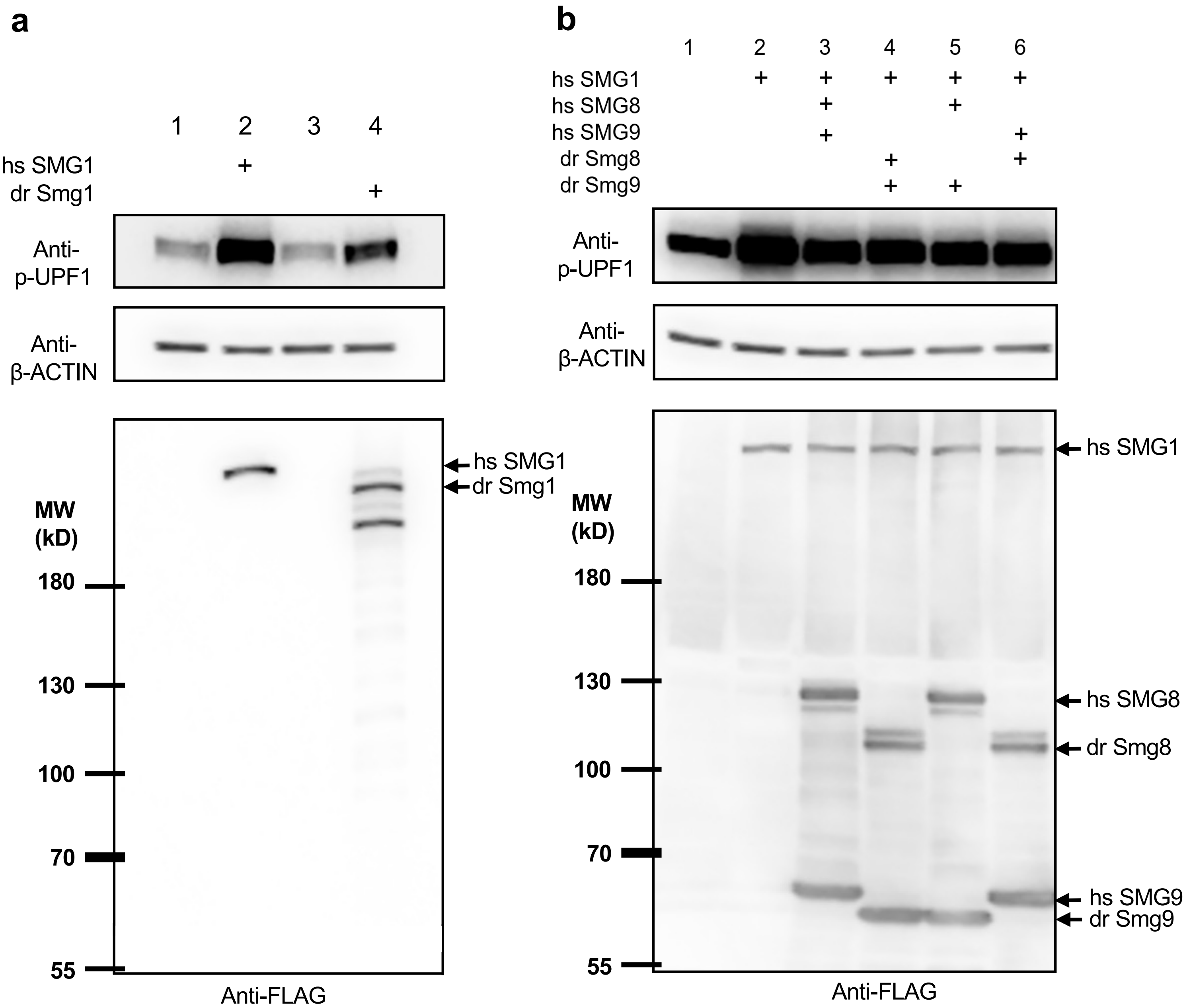

Supplementary Figure 10. Components of the SMG1:SMG8:SMG9 complex are interchangeable between humans and zebrafish. (a) Western blot analysis of human UPF1 phosphorylation following overexpression of human SMG1 (lane 2) and zebrafish Smg1 (lane 4). (b) Western blot analysis of human UPF1 phosphorylation following the overexpression of human and zebrafish components of the SMG1:SMG8:SMG9 complex. hs: *Homo sapiens*; dr: *Danio rerio*.

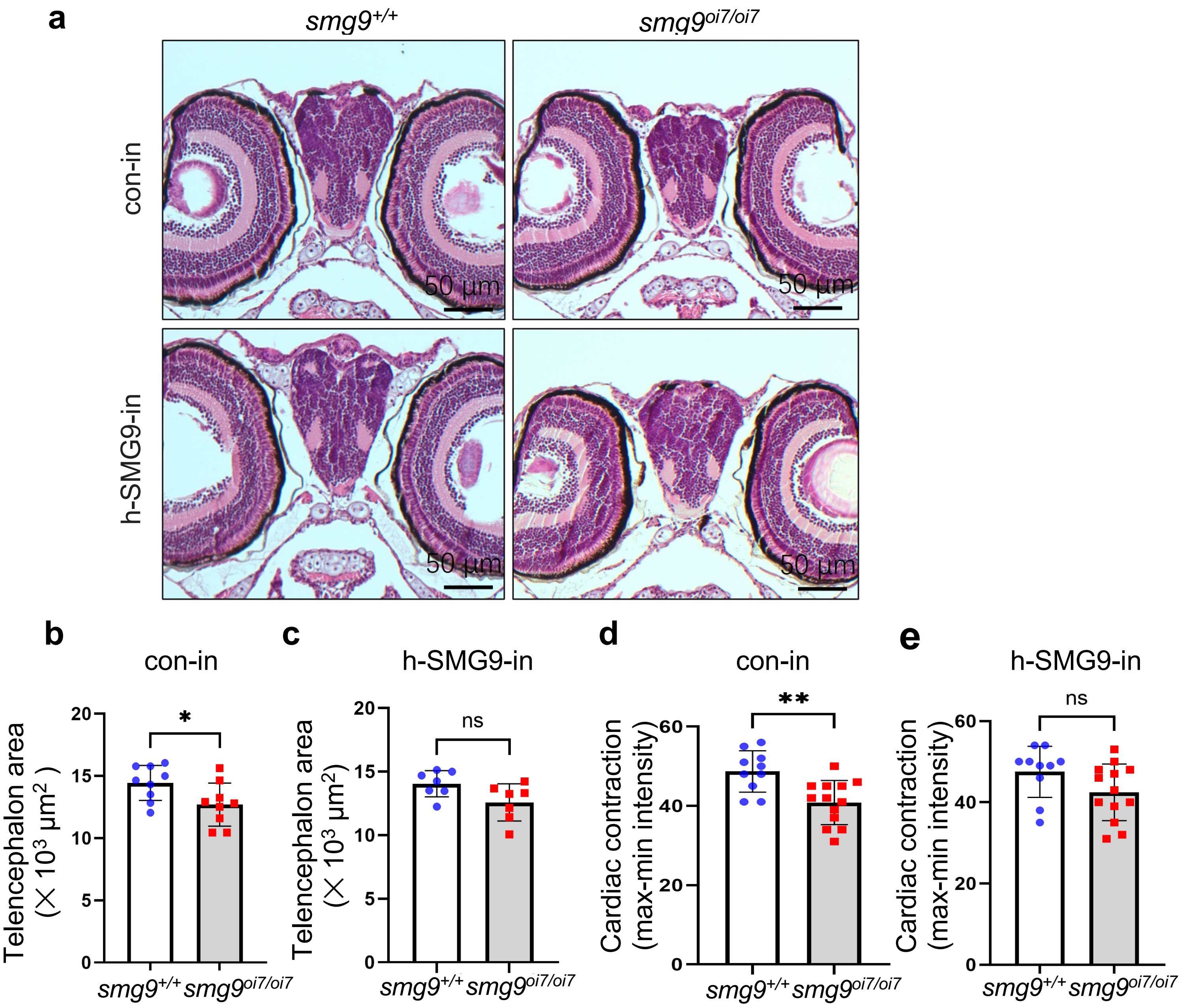

Supplementary Figure 11. Comparison of brain size and cardiac contraction after human SMG9 mRNA injection (a) Representative images of H&E staining of human *SMG9* mRNA-injected (h-SMG9-in) and control-injected (con-in) larvae at 14 dpf. Scale bar: 50 µm. (**b**) Quantification of the telencephalon area of control-injected (con-in) larvae at 14 dpf by H&E staining. **(c)** Quantification of telencephalon area of human *SMG9* mRNA-injected (h-SMG9-in) larvae at 14 dpf using H&E staining. **(d)** Quantification of cardiac contractions in the control-injected (con-in) larvae at 6 dpf. **(e)** Quantification of cardiac contractions in h-SMG9-in larvae at 6 dpf. $*P < 0.05$, $**P < 0.01$. ns: not significant.

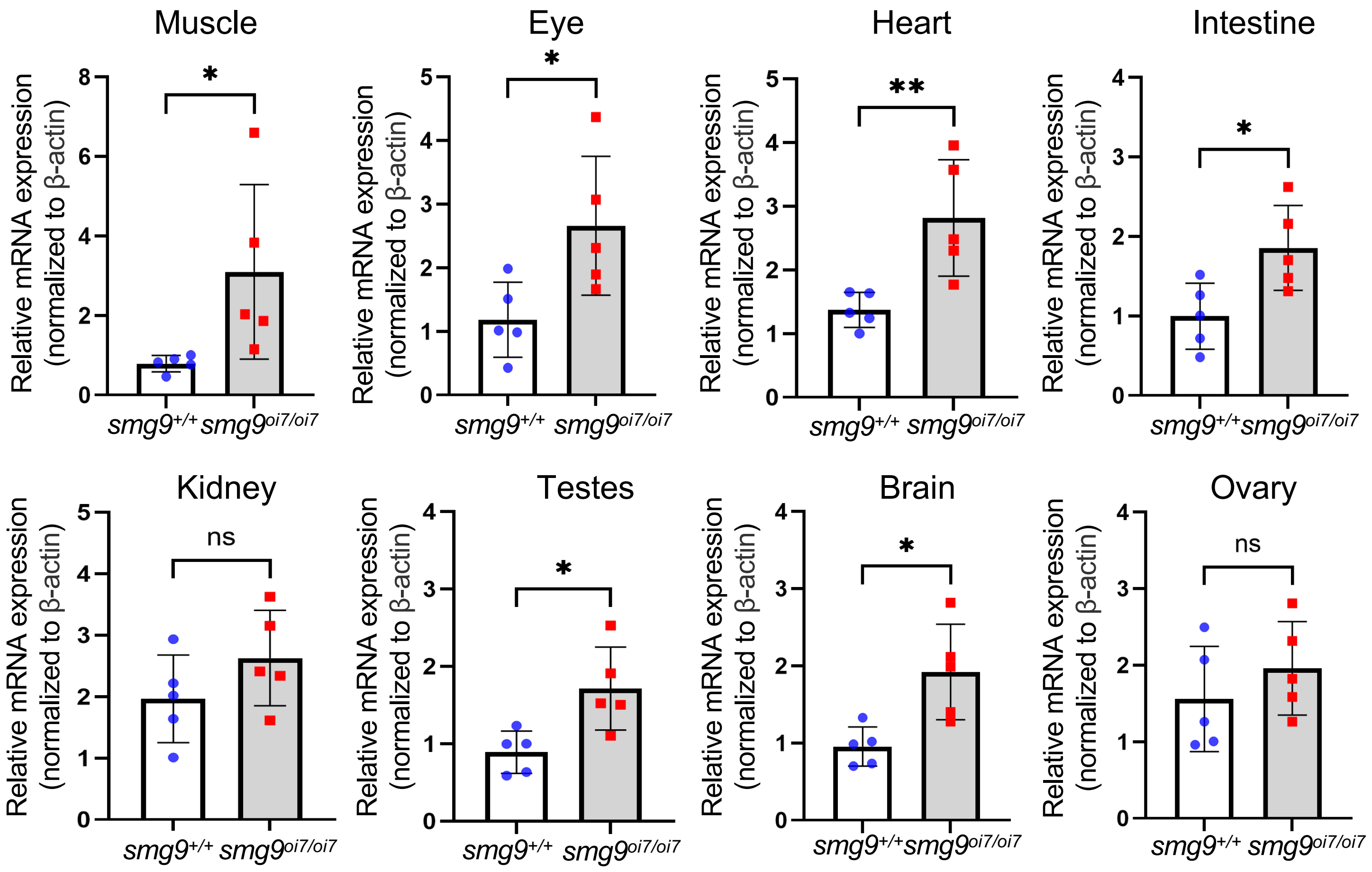

Supplementary Figure 12. RT-qPCR analysis of *smox* mRNA expression in various tissues of *smg9*$^{+/+}$ and *smg9*$^{oi7/oi7}$ zebrafish at 6 mpf. Error bars indicate SD. **P* < 0.05, ***P* < 0.01, and ****P* < 0.001. ns: not significant.

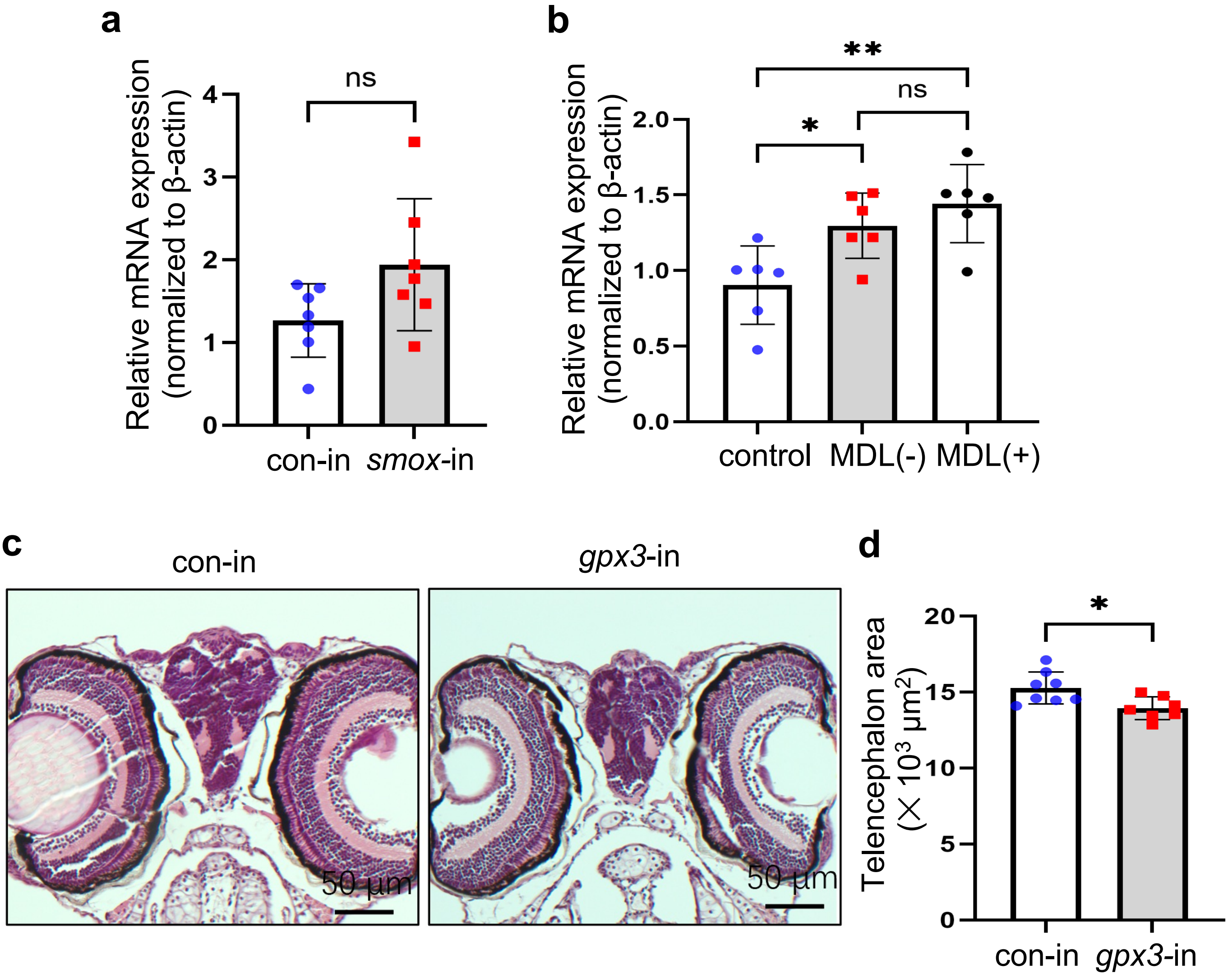

Supplementary Figure 13. Smox does not affect the upregulation of *gpx3* mRNA. (a) The mRNA levels of gpx3 in *smox* mRNA-injected (*smox*-in) zebrafish compared to control mRNA-injected (con-in) zebrafish. (b) The mRNA level of *gpx3* in 7 dpf zebrafish treated with or without the SMOX inhibitor MDL72527. (c) Representative H&E staining images of *gpx3*-in and con-in larvae telencephalons at 14 dpf. Scale bar: 50 μm. (d) Quantification of the telencephalon area of *gpx3*-in and con-in larvae at 14 dpf. *$P < 0.05$. ns: not significant.

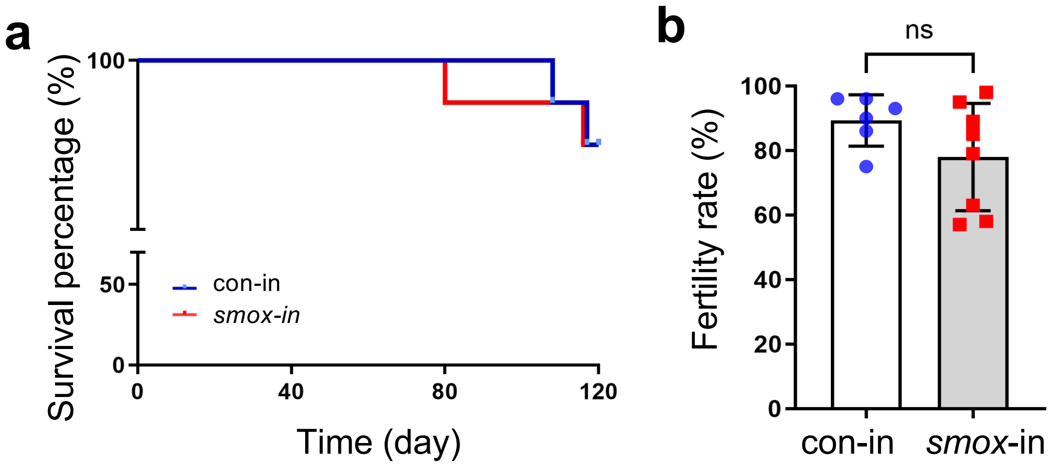

Supplementary Figure 14. Injection of *smox* mRNA (*smox*-in) and control (con-in) into zebrafish. (**a**) Kaplan–Meier survival curve analysis showing the lifespan of *smox*-in and con-in zebrafish in each group (n =20). (**b**) Fertility rate of con-in and *smox*-in zebrafish at 3 mpf, as determined by mating con-in and *smox*-in male zebrafish with wild-type female zebrafish and counting the embryo survival. Error bars indicate SD. ns: not significant.

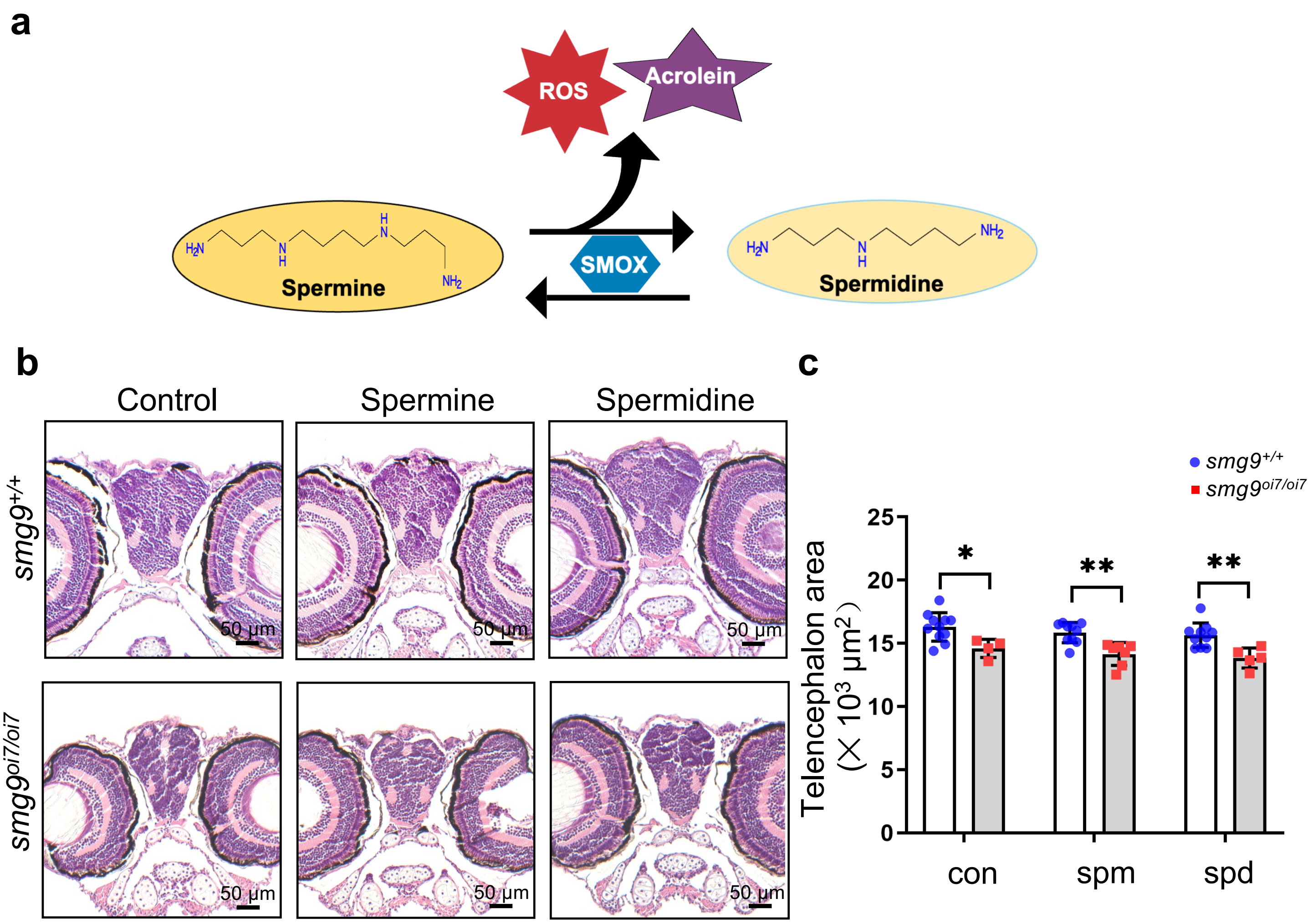

Supplementary Figure 15. Spermidine and spermine do not affect *smg9*$^{-/-}$ zebrafish. (a) Schematic of the mechanism of SMOX catalyzing the oxidation of spermine to spermidine with the production of ROS and acrolein. **(b)** Representative images of H&E staining of the telencephalon of *smg9*$^{+/+}$ and *smg9*$^{oi7/oi7}$ larvae at 14 dpf after treatment with or without spermidine or spermine. Scale bar: 50 μm. **(c)** Quantification of the telencephalon area of *smg9*$^{+/+}$ and *smg9*$^{oi7/oi7}$ larvae 14 dpf after treatment with or without spermidine or spermine. Error bars indicate SD. con: control; spm: spermine; spd: spermidine. *$P < 0.05$, **$P < 0.01$.

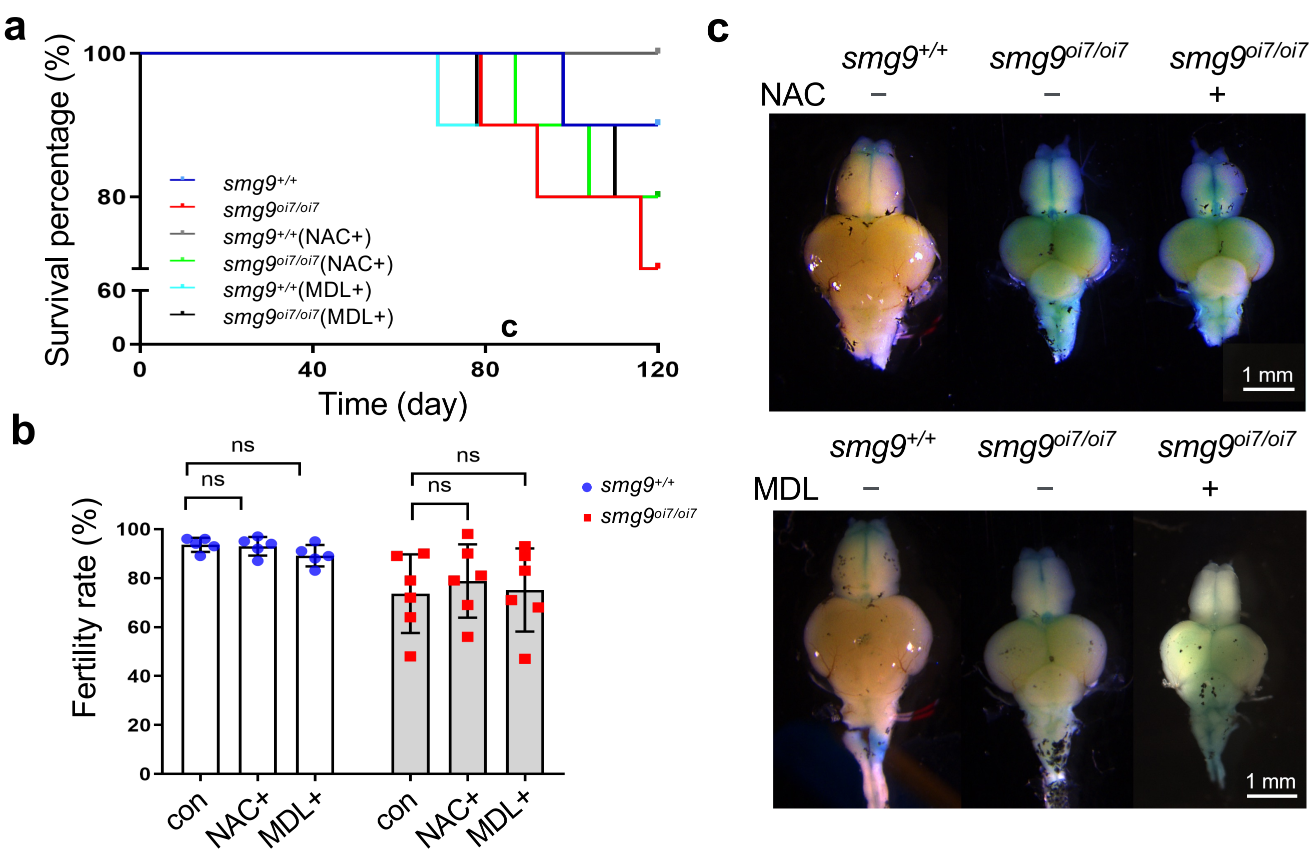

Supplementary Figure 16. ROS and SMOX inhibitors do not affect the aging phenotype in *smg9*$^{oi7/oi7}$ zebrafish. *smg9*$^{+/+}$ and *smg9*$^{oi7/oi7}$ zebrafish were treated with or without N-acetylcysteine (NAC) or MDL72527 (MDL) for 14 dpf. **(a)** Kaplan–Meier survival curve analysis showing the lifespan of each group (n = 10). **(b)** Fertility rate of *smg9*$^{+/+}$ and *smg9*$^{oi7/oi7}$ zebrafish at 3 mpf, as determined by mating *smg9*$^{+/+}$ and *smg9*$^{oi7/oi7}$ male zebrafish with *smg9*$^{+/+}$ female zebrafish and counting embryo survival. **(c)** SA-β-gal staining of the brains of *smg9*$^{+/+}$ and *smg9*$^{oi7/oi7}$ zebrafish at 4 mpf. Scale bar: 1 mm.

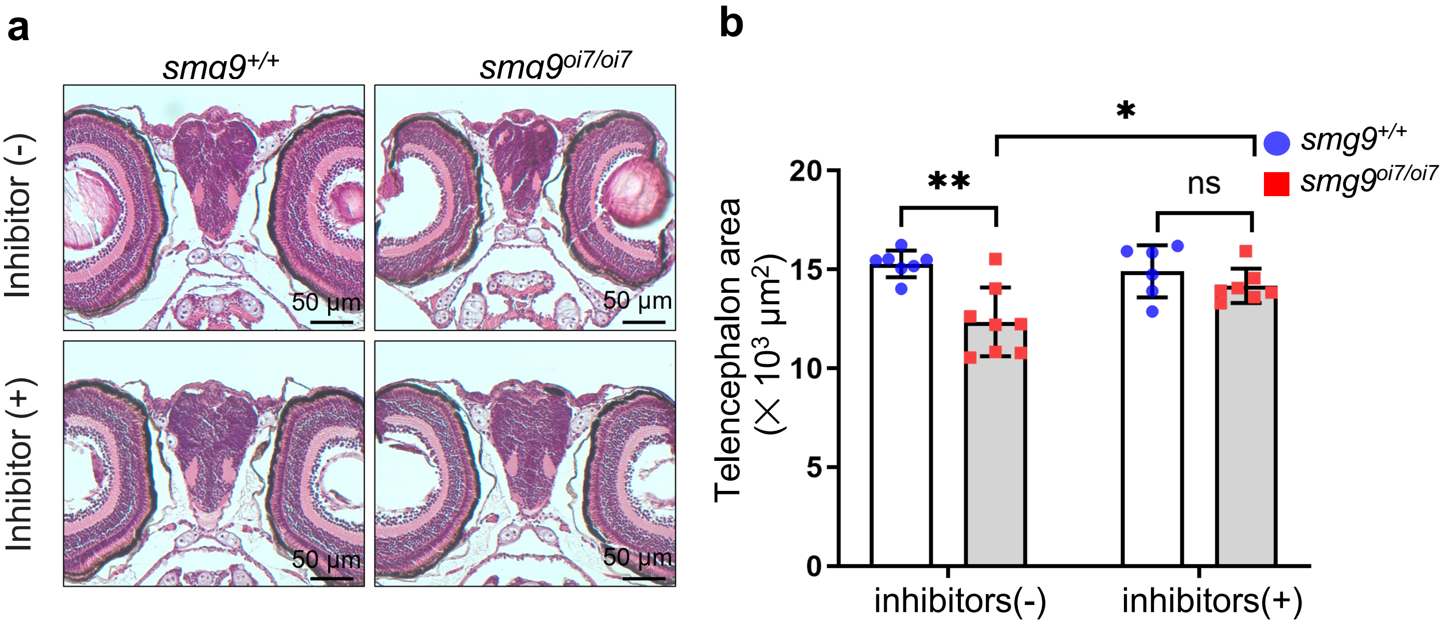

Supplementary Figure 17. Co-treatment with SMOX and ROS inhibitors for smg9oi7/oi7 larvae. (a) H&E staining of brain tissues at 14 dpf, treated or untreated with both SMOX inhibitor (MDL72527) and ROS inhibitor (NAC) for 3 days from 3 h post-fertilization. (b) Significant improvement in brain malformations in *smg9*$^{oi7/oi7}$ larvae after co-treatment with SMOX and ROS inhibitors. *$P < 0.05$, **$P < 0.01$. ns: not significant.

Uncropped and unedited gel images related to Figure 1b.

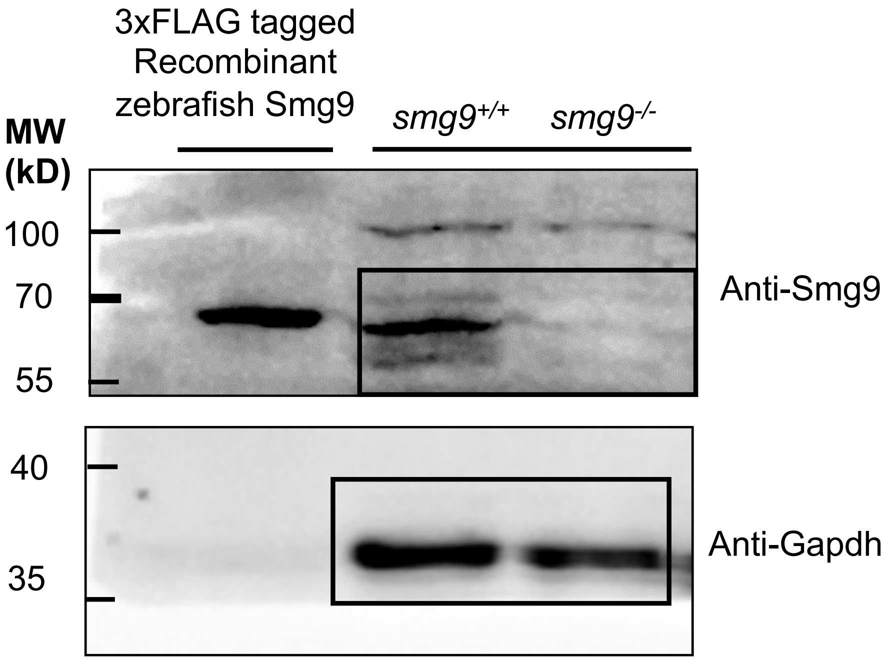

Uncropped and unedited gel images related to Supplementary Figure 10.

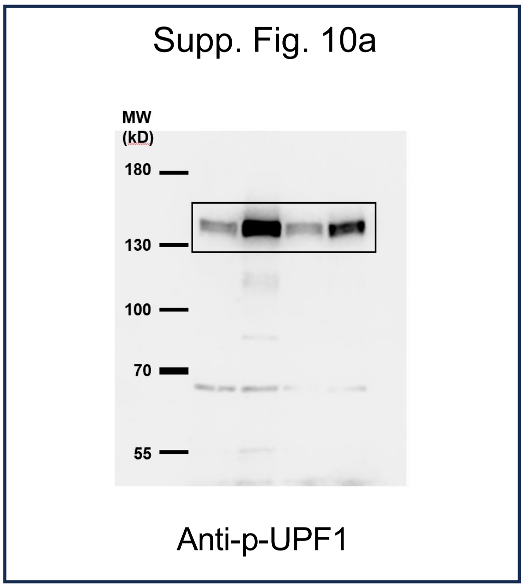

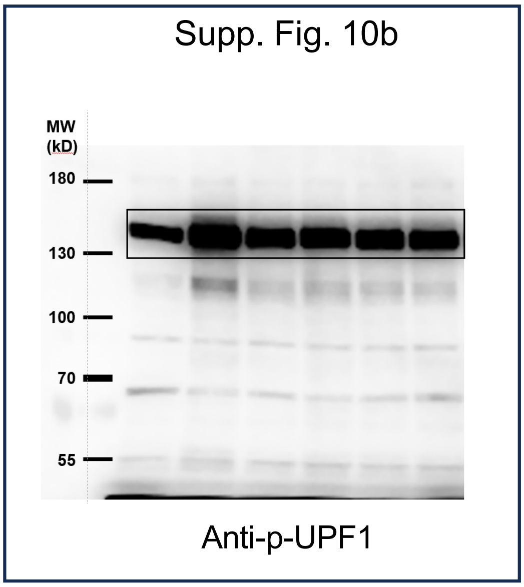

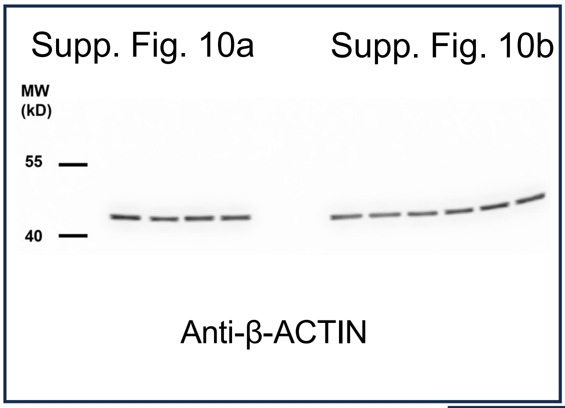

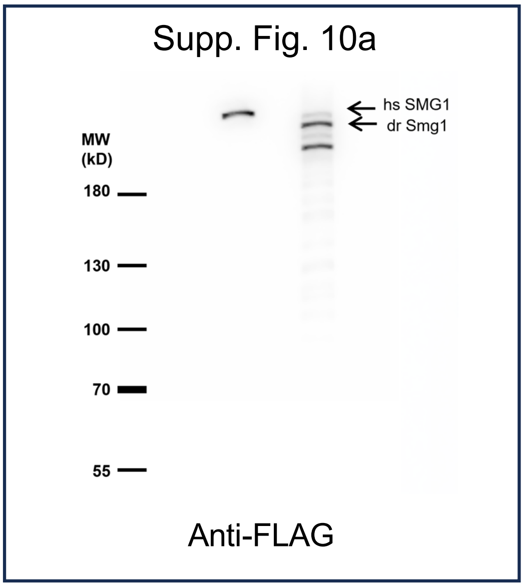

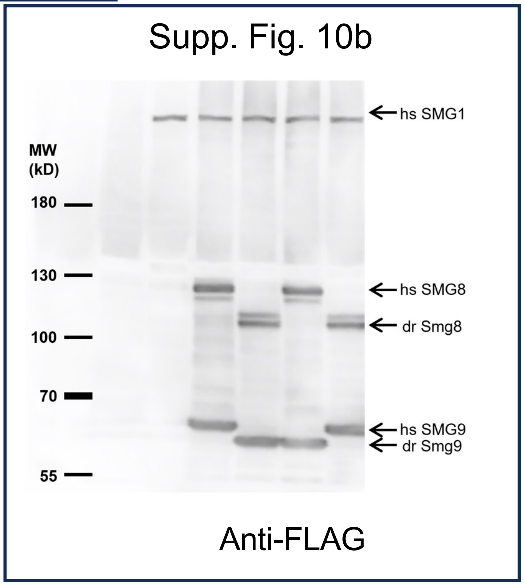

Uncropped and unedited gel images related to Figure 5k.

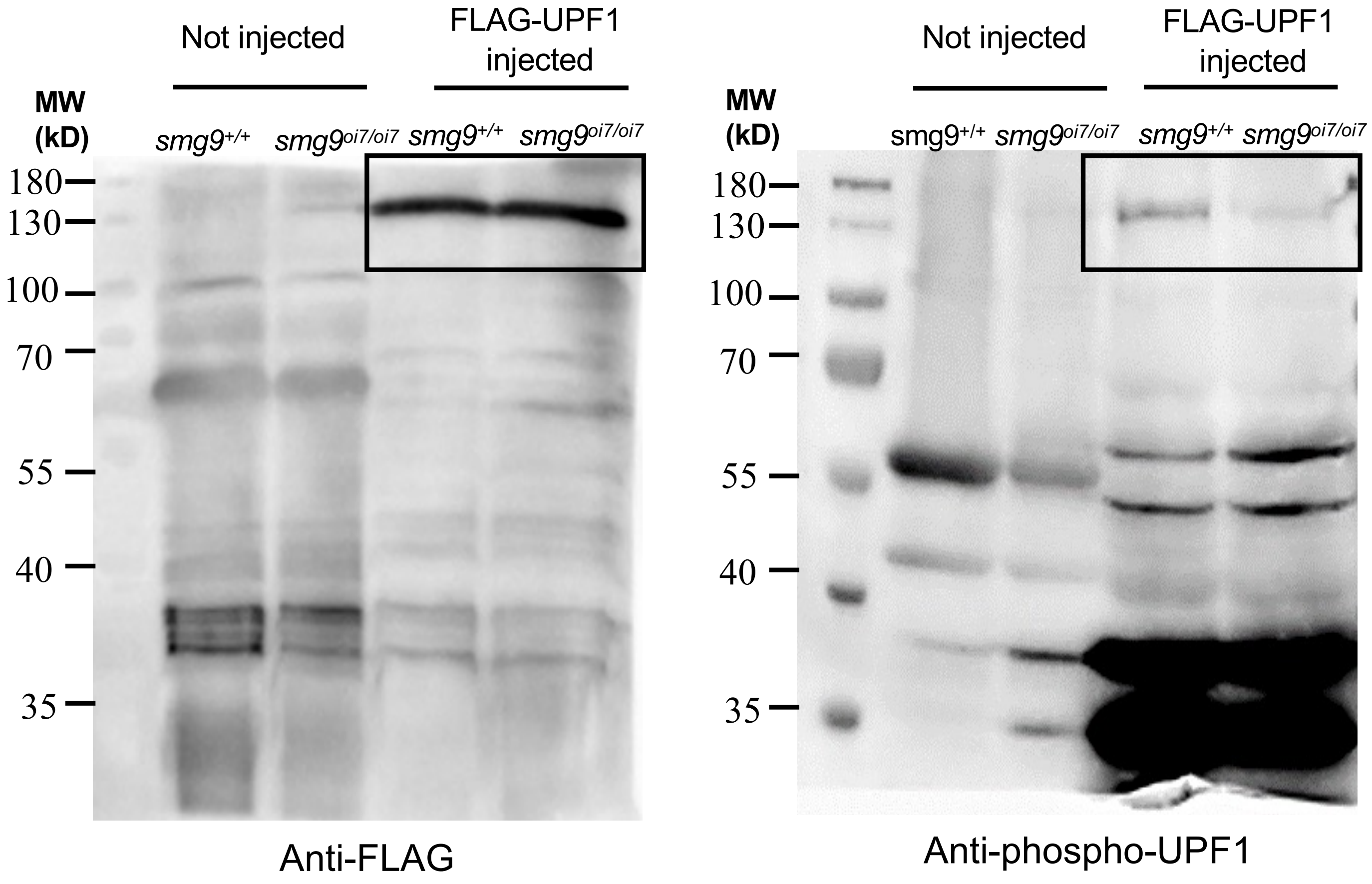

Supplement: Supplementary file 2 — Supplementary information [file 42003_2024_6356_MOESM2_ESM.pdf]
