# Supplementary material for: Effect of nonsense-mediated mRNA decay factor SMG9 deficiency on premature aging in zebrafish
Source: Commun Biol. 2024 May 28;7:654. doi: 10.1038/s42003-024-06356-6 (PMC11133409; doi:10.1038/s42003-024-06356-6)
Supplement: Supplementary file 4 — Reporting summary [file 42003_2024_6356_MOESM4_ESM.pdf]

nature portfolio

Corresponding author(s): Toshikatsu Hanada
Last updated by author(s): May 2, 2024

Reporting Summary

Nature Portfolio wishes to improve the reproducibility of the work that we publish. This form provides structure for consistency and transparency in reporting. For further information on Nature Portfolio policies, see our Editorial Policies and the Editorial Policy Checklist.

Statistics

For all statistical analyses, confirm that the following items are present in the figure legend, table legend, main text, or Methods section.

n/a	Confirmed	
☐	☒	The exact sample size (*n*) for each experimental group/condition, given as a discrete number and unit of measurement
☐	☒	A statement on whether measurements were taken from distinct samples or whether the same sample was measured repeatedly
☐	☒	The statistical test(s) used AND whether they are one- or two-sided<br>*Only common tests should be described solely by name; describe more complex techniques in the Methods section.*
☒	☐	A description of all covariates tested
☒	☐	A description of any assumptions or corrections, such as tests of normality and adjustment for multiple comparisons
☐	☒	A full description of the statistical parameters including central tendency (e.g. means) or other basic estimates (e.g. regression coefficient) AND variation (e.g. standard deviation) or associated estimates of uncertainty (e.g. confidence intervals)
☐	☒	For null hypothesis testing, the test statistic (e.g. *F*, *t*, *r*) with confidence intervals, effect sizes, degrees of freedom and *P* value noted<br>*Give P values as exact values whenever suitable.*
☒	☐	For Bayesian analysis, information on the choice of priors and Markov chain Monte Carlo settings
☒	☐	For hierarchical and complex designs, identification of the appropriate level for tests and full reporting of outcomes
☒	☐	Estimates of effect sizes (e.g. Cohen's *d*, Pearson's *r*), indicating how they were calculated

Our web collection on statistics for biologists contains articles on many of the points above.

Software and code

Policy information about availability of computer code

Data collection	Zebrafish behavioral tests were performed using the Pro Stream Webcam C922n (Logicool), recorded in vivo videos using fluorescence stereo microscope (Biorevo BZ-9000, KEYENCE), hematoxylin and eosin (HE),masson trichrome staining and Immunofluorescence staining using Axio Imager M2 microscope (Carl Zeiss, Jena, Germany).SA-β-gal staining and Acrolein RED (FDV-0022, Funakoshi) staining using a Leica M205 FA fluorescent stereo microscope (Leica, Wetzlar, Germany),TEM using transmission electron microscope (H-7650; Hitachi, Tokyo, Japan).Western blot using Fusion CAPT Advance Software Version 17.02,RT-qPCR was using LightCycler 96 system (Roche)
Data analysis	imageJ(image processing), GraphPad Prism 8(graph and statistical analysis), G*Power software 3.1(statistical analysis), ZantiksMWP(locomotion analysis), FlowJo (flow cytometer data analysis),Excel 2019(statistical anlysis)

For manuscripts utilizing custom algorithms or software that are central to the research but not yet described in published literature, software must be made available to editors and reviewers. We strongly encourage code deposition in a community repository (e.g. GitHub). See the Nature Portfolio guidelines for submitting code & software for further information.

Data

Policy information about availability of data

All manuscripts must include a data availability statement. This statement should provide the following information, where applicable:

- Accession codes, unique identifiers, or web links for publicly available datasets
- A description of any restrictions on data availability
- For clinical datasets or third party data, please ensure that the statement adheres to our policy

Data generated or analyzed in the present study are included in this published article and its supplementary information files. Further information and requests should be directed to and will be fulfilled by the corresponding author.

Research involving human participants, their data, or biological material

Policy information about studies with human participants or human data. See also policy information about sex, gender (identity/presentation), and sexual orientation and race, ethnicity and racism.

Reporting on sex and gender	*Use the terms sex (biological attribute) and gender (shaped by social and cultural circumstances) carefully in order to avoid confusing both terms. Indicate if findings apply to only one sex or gender; describe whether sex and gender were considered in study design; whether sex and/or gender was determined based on self-reporting or assigned and methods used. Provide in the source data disaggregated sex and gender data, where this information has been collected, and if consent has been obtained for sharing of individual-level data; provide overall numbers in this Reporting Summary. Please state if this information has not been collected. Report sex- and gender-based analyses where performed, justify reasons for lack of sex- and gender-based analysis.*
Reporting on race, ethnicity, or other socially relevant groupings	*Please specify the socially constructed or socially relevant categorization variable(s) used in your manuscript and explain why they were used. Please note that such variables should not be used as proxies for other socially constructed/relevant variables (for example, race or ethnicity should not be used as a proxy for socioeconomic status). Provide clear definitions of the relevant terms used, how they were provided (by the participants/respondents, the researchers, or third parties), and the method(s) used to classify people into the different categories (e.g. self-report, census or administrative data, social media data, etc.) Please provide details about how you controlled for confounding variables in your analyses.*
Population characteristics	*Describe the covariate-relevant population characteristics of the human research participants (e.g. age, genotypic information, past and current diagnosis and treatment categories). If you filled out the behavioural & social sciences study design questions and have nothing to add here, write "See above."*
Recruitment	*Describe how participants were recruited. Outline any potential self-selection bias or other biases that may be present and how these are likely to impact results.*
Ethics oversight	*Identify the organization(s) that approved the study protocol.*

Note that full information on the approval of the study protocol must also be provided in the manuscript.

Field-specific reporting

Please select the one below that is the best fit for your research. If you are not sure, read the appropriate sections before making your selection.

☒ Life sciences ☐ Behavioural & social sciences ☐ Ecological, evolutionary & environmental sciences

For a reference copy of the document with all sections, see nature.com/documents/nr-reporting-summary-flat.pdf

Life sciences study design

All studies must disclose on these points even when the disclosure is negative.

Sample size	Samples were taken from at least three repeat experiments. Samples were determined according to a pilot study as well as on the basis of previous experimental experience.
Data exclusions	Unviable embryos were excluded from our analyses. Missed injected embryos were also excluded from analysis.
Replication	Each experiment was performed with at least 3 biological replicates. All the reported results are from experiments in which each repeat gave similar results.
Randomization	All samples were randomly allocated into experimental groups.
Blinding	No formal blinding was used.

Reporting for specific materials, systems and methods

We require information from authors about some types of materials, experimental systems and methods used in many studies. Here, indicate whether each material, system or method listed is relevant to your study. If you are not sure if a list item applies to your research, read the appropriate section before selecting a response.

Materials & experimental systems

n/a	Involved in the study	
☐	☒	Antibodies
☐	☒	Eukaryotic cell lines
☒	☐	Palaeontology and archaeology
☐	☒	Animals and other organisms
☒	☐	Clinical data
☒	☐	Dual use research of concern
☒	☐	Plants

Methods

n/a	Involved in the study	
☒	☐	ChIP-seq
☐	☒	Flow cytometry
☒	☐	MRI-based neuroimaging

Antibodies

Antibodies used	SMG9 (A302-211A, Bethyl Laboratories), UPF1 (F1804, Sigma-Aldrich), phospho-UPF1 (Millipore, #07-1016), Cleaved Caspase-3 (D175; cell signaling), S-100 (IR504; Dako), PCNA (M0879; Dako), HuC/HuD (A21271; Invitrogen), and phospho-H2A.X (92590, Cell Signaling). Secondary antibodies used were Alexa Fluor 488 (A21206; Invitrogen) and Alexa Fluor 555 (A21422; Invitrogen).
Validation	All antibodies were validated by their source company. The antibodies used in this study have been previously described and used in previously published research.

Eukaryotic cell lines

Policy information about cell lines and Sex and Gender in Research

Cell line source(s)	293T cel line: originally derived from human embryonic kidney cells (HEK 293) that were transformed with a modified adenovirus.
Authentication	293T cell line: Morphology and proliferation rate monitoring of the 293T cell line.
Mycoplasma contamination	We confirmed that our 293T cell line was free of mycoplasma contamination.
Commonly misidentified lines (See ICLAC register)	The only cell line we used was the 293T cell line, which has not been misidentified.

Animals and other research organisms

Policy information about studies involving animals; ARRIVE guidelines recommended for reporting animal research, and Sex and Gender in Research

Laboratory animals	Zebrafish wild-type(AB strain; ZFIN, Eugene, OR, USA) ,Tg(cmlc2-EGFP).<br>smg9 and smg8 mutants were generated in our laboratory using CRISPR-Cas9 System (Integrated DNA Technologies, Coralville, IA, USA).
Wild animals	This study did not involve field-collected asmples.
Reporting on sex	In the experiments with embryos and larvae, sex was not considered. In experiments with adult fish, males were used.
Field-collected samples	This study did not involve field-collected samples.
Ethics oversight	All experimental procedures were performed according to institutional and national guidelines and regulations. The study protocol was approved by Oita University (approval number:180506).

Note that full information on the approval of the study protocol must also be provided in the manuscript.

Plants

Seed stocks

Report on the source of all seed stocks or other plant material used. If applicable, state the seed stock centre and catalogue number. If plant specimens were collected from the field, describe the collection location, date and sampling procedures.

Novel plant genotypes

Describe the methods by which all novel plant genotypes were produced. This includes those generated by transgenic approaches, gene editing, chemical/radiation-based mutagenesis and hybridization. For transgenic lines, describe the transformation method, the number of independent lines analyzed and the generation upon which experiments were performed. For gene-edited lines, describe the editor used, the endogenous sequence targeted for editing, the targeting guide RNA sequence (if applicable) and how the editor was applied.

Authentication

Describe any authentication procedures for each seed stock used or novel genotype generated. Describe any experiments used to assess the effect of a mutation and, where applicable, how potential secondary effects (e.g. second site T-DNA insertions, mosiacism, off-target gene editing) were examined.

Flow Cytometry

Plots

Confirm that:

☒ The axis labels state the marker and fluorochrome used (e.g. CD4-FITC).

☒ The axis scales are clearly visible. Include numbers along axes only for bottom left plot of group (a 'group' is an analysis of identical markers).

☒ All plots are contour plots with outliers or pseudocolor plots.

☒ A numerical value for number of cells or percentage (with statistics) is provided.

Methodology

Sample preparation

Brains dissected at 3 and 6 mpf were trypsinized to detach the cells. The collected cells were incubated with CellROX for 3 min in the dark and then analyzed for cellular fluorescence intensity.

Instrument

FACS LSR Fortessa X-20 (5-LASER)

Software

FlowJo V10 software(Flowjo, LLC)

Cell population abundance

No sorting was performed

Gating strategy

Cells were gated on FSC-A/SSC-A

☒ Tick this box to confirm that a figure exemplifying the gating strategy is provided in the Supplementary Information.
